# 5*α*,6*α*-Epoxyphytosterols and 5*α*,6*α*-Epoxycholesterol Increase Nitrosative Stress and Inflammatory Cytokine Production in Rats on Low-Cholesterol Diet

**DOI:** 10.1155/2020/4751803

**Published:** 2020-06-08

**Authors:** Tomasz Wielkoszyński, Jolanta Zalejska-Fiolka, Joanna K. Strzelczyk, Aleksander J. Owczarek, Armand Cholewka, Katarzyna Kokoszczyk, Agata Stanek

**Affiliations:** ^1^Higher School of Strategic Planning in Dąbrowa Górnicza, Kościelna 6 St., 41-300 Dąbrowa Górnicza, Poland; ^2^Wielkoszyński Medical Center and Laboratory, Piłsudskiego 10 St., 41-300 Dąbrowa Górnicza, Poland; ^3^Medical University of Silesia, Faculty of Medical Sciences in Zabrze, Department of Biochemistry, Jordana 19 St., 41-808 Zabrze, Poland; ^4^Medical University of Silesia, Faculty of Medical Sciences in Zabrze, Department of Medical and Molecular Biology, Jordana 19 St., 41-808 Zabrze, Poland; ^5^Medical University of Silesia, Faculty of Pharmaceutical Sciences, Department of Statistics, Department of Instrumental Analysis, Ostrogórska 30 St., Sosnowiec 41-209, Poland; ^6^University of Silesia, Faculty of Science and Technology, Katowice, Bankowa St., 40-007 Katowice, Poland; ^7^Medical University of Silesia, Faculty of Medical Sciences in Zabrze, Department of Internal Medicine, Angiology and Physical Medicine, Batorego 15 St., 41-902 Bytom, Poland

## Abstract

**Objective:**

Oxidized cholesterol derivatives are compounds with proven atherogenic and mutagenic effects. However, little is known about the effect of oxidized plant sterol derivatives (oxyphytosterols), whose structure is similar to the one of oxycholesterols. Our previous studies indicate that they have a similar profile of action, e.g., both exacerbate disorder of lipid metabolism and oxidative stress in experimental animals. The aim of the present study was to assess the effect of epoxycholesterol and epoxyphytosterols (mainly sitosterol) on the severity of nitrosative stress and the concentration of selected proinflammatory cytokines in blood and liver tissue of rats on a low-cholesterol diet. *Material and Methods*. Forty-five male Wistar rats were fed with feed containing 5*α*,6*α*-epoxyphytosterols (ES group, *n*: 15), 5*α*,6*α*-epoxycholesterol (ECh group, *n*: 15), and oxysterol-free feed (C group, *n*: 15) for 90 days (daily dose of oxysterols: 10 mg/kg). At the end of the experiment, nitrotyrosine, TNF-*α*, IL-1*β*, IL-6, and lipid metabolism parameters were determined in blood serum. Furthermore, nitrotyrosine, TNF-*α*, cholesterol, and triglyceride content were determined in liver homogenates.

**Results:**

Serum nitrotyrosine, IL-1*β*, and TNF-*α* concentrations as well as TNF-*α* content in the liver were significantly higher in both groups exposed to oxysterols (ECh and ES groups) as compared to the C group. The serum IL-6 level and nitrotyrosine content in the liver were significantly higher in the ECh group, as compared to the C and ES groups. There was evidence to support the dyslipidemic effect of studied compounds.

**Conclusions:**

The results indicate that oxidized plant sterols have a similar toxicity profile to that of oxycholesterols, including nitrosative stress induction, proinflammatory effect, and impaired lipid metabolism.

## 1. Introduction

Oxysterols have been implicated in the underlying mechanisms of inflammation-mediated diseases, such as atherosclerosis, neurodegenerative disorders, and cancer [1–4]. Through upregulation of NADPH oxidase (NOX) family enzymes [2], which are the main source of cellular reactive oxygen species (ROS) [5], oxysterols may cause extensive ROS generation, causing oxidative and nitrosative stress. Reactive nitrogen species act together with ROS causing cellular damage. Nitrotyrosine is one of the nitrosative stress markers. The cytokine system plays the crucial role in triggering nitrosative stress [6].

Apart from endogenous production, oxycholesterols may also be sourced from nutrition, in particular from cholesterol-rich foods undergoing long-term thermal processing and exposed to gamma irradiation or long-term storage [7].

Thus, the aim of the research was to assess the effect of 5*α*,6*α*-epoxyphytosterols and 5*α*,6*α*-epoxycholesterol on nitrosative stress and cytokine system in experimental animals.

## 2. Material and Methods

### 2.1. Animals

The protocol was approved by the Bioethical Committee for Animal Experimentation of the Medical University of Silesia in Katowice, Poland (approval no. 27/2007, dated April 17, 2007). All animals received humane care in compliance with the 8th edition of the *Guide for the Care and Use of Laboratory Animals* published by the National Institutes of Health [8].

Male Wistar rats, with the body weight of 130-180 g at baseline, were sourced from the Center for Experimental Medicine, Medical University of Silesia, in Katowice. During the experiment, the rats were kept on wood shaving bedding in standard single rodent cages, at the temperature of 20-25°C, with artificial lighting (a 12 h/12 h day/night cycle). The feed was administered once a day, and tap water was available ad libitum. Prior to the commencement of the experiment, the animals were kept in the conditions described above for an acclimation period of 2 weeks to ensure reproducible results.

The rats were divided into 3 groups (15 animals each), to receive the following:

- Feed containing 5*α*,6*α*-epoxyphytosterol acetate at 100 mg per 1 kg of feed (ES group)
- Feed containing 5*α*,6*α*-epoxycholesterol acetate at 100 mg per 1 kg of feed (ECh group)
- Oxysterol-free feed (controls, C group)

Daily estimated sterol dose was 10 mg per 1 kg of animal body weight (assuming the feed intake equal to 10% of animal body weight). Labofeed B (Wytwórnia Pasz, Kcynia, Poland), a standard laboratory maintenance feed for rodents, was used during the study. The feed was administered for 90 days. The animals were weighted before and after the experiment. After 3 months, the rats were anaesthetised with the mixture of ketamine (50 mg/kg), droperidol (1 mg/kg), and fentanyl (0.1 mg/kg) administered i.m. and euthanized by cardiac exsanguination and cervical dislocation.

### 2.2. Synthesis of 5*α*,6*α*-Epoxycholesterol and 5*α*,6*α*-Epoxyphytosterol Acetate

5*α*,6*α*-Epoxycholesterol acetate and 5*α*,6*α*-epoxyphytosterol acetate were synthetized from cholesterol and sitosterol (Sigma-Aldrich, USA), respectively, by acetylation and subsequent oxidation with m-chlorperoxybenzoic acid (Sigma-Aldrich, USA) as described by McCarthy [9]. Next, the oxidation mixture was purified by column chromatography on silica gel using chloroform-acetone (4 : 1, *v*/*v*) as a mobile phase. Fractions containing pure ester were controlled by TLC technique (silica gel plates, solvent as above), pooled, and dried under vacuum. As per manufacturer's data sheet, sitosterol contains about 90% *β*-sitosterol and ca. 10% other phytosterols and phytostanols. Thus, its oxidation products were termed 5*α*,6*α*-epoxyphytosterols.

### 2.3. Blood Sample Collection

Blood samples were collected into serum clot activator tubes (Sarstedt, S-Monovette). Directly after centrifugation (900g for 10 min. at 4°C), serum samples were separated and stored at –70°C, until biochemical analyses were performed [10–12].

### 2.4. Liver Homogenate Preparation

After the rats were euthanized, their livers were quickly excised, weighed, and homogenised in cooled (4°C) PBS at 1 : 10. The prepared 10% homogenates were frozen at -70°C for further analyses.

Full homogenates were used for lipid determination, whereas the supernatant obtained with centrifugation (10 min., 20 000 rpm, 4°C) of defrosted homogenates was used for nitrotyrosine and TNF-alpha determinations.

The total cholesterol, triglycerides, nitrotyrosine, and TNF-*α* concentrations were assayed both in serum samples and liver homogenates. Additionally, we determined serum IL-1, IL-6, and LDL- and HDL-cholesterol levels.

### 2.5. Biochemical Analyses

#### 2.5.1. Serum Lipid Determination

Total cholesterol and triglyceride concentrations were assayed in serum samples using a standard enzymatic method (Emapol, Poland). HDL-cholesterol was determined using an enzymatic method after precipitation of other lipoproteins with phosphotungstic acid (Emapol, Poland). For the LDL-cholesterol assay, a Quantolip LDL kit (Technoclone, Austria) was used. Total phospholipids in serum were assayed with the use of an enzymatic method (DiaSys GmbH, Germany). All analyses were performed using the EM280 biochemical analyzer (Emapol, Poland). Interassay and intra-assay coefficients of variation (CV) were below 3% and 5%, respectively, for all parameters.

#### 2.5.2. Cholesterol and Triglycerides in Liver Homogenates

Tissue lipids were extracted by mixing 1 volume of 10% liver homogenate prepared in phosphate-buffered solution (PBS), with 9 volumes of isopropyl alcohol. After 24 hours, the supernatant was separated from the protein precipitate by centrifugation, and 1 mL of the clear supernatant was collected to two glass tubes.

In order to determine the *cholesterol* concentration, the solvent was dried under reduced pressure and the dry residue was dissolved in 0.1 mL of Triton X-100-methoxyethanol (2 : 8, *v*/*v*) mixture. After, 1 mL of cholesterol reagent (Human, Germany) was added, the samples were incubated in a water bath at 37°C for 30 minutes with continuous shaking, and the absorbance was measured at 510 nm against a blank. The calibration solution was cholesterol dissolved in the Triton X-100-methoxyethanol (2 : 8, *v*/*v*) mixture.

To determine *triglyceride* concentrations, the dry residue in the second tube was dissolved in 1 mL of Triton X100-methoxyethanol mixture (2 : 8, *v*/*v*) and the determination was performed on the Technicon RA-XT analyzer according to the standard serum processing procedure.

All results were expressed as mg/g (wet matter basis).

#### 2.5.3. Determination of Inflammatory Cytokines

The concentration of TNF-*α*, IL-6, and IL-1*β* in rat serum and TNF-*α* in rat liver homogenates was determined by ELISA using Diaclone kits (France): rat TNF-*α* EliPair, cat. no. 872.010.010; murine IL-6 EliPair, cat. no. 861.020.010; and rat IL-1*β* ELISA kit, cat. no. 670.040.192 according to the manufacturer's instructions. The absorbance was measured using the PowerWave XS microplate reader (BioTek, USA), and data was processed using the KC Junior software bundle (BioTek, USA). The within-run coefficient of variation for the TNF-*α*, IL-1*β*, and IL-6 determination was 5.2%, 7.1%, and 4.8%, respectively.

The results of TNF-*α* concentration in liver homogenates were expressed as pg/mg total protein determined using the Lowry method [13]. Serum TNF-*α*, IL-1*β*, and IL-6 results were expressed as pg/mL.

#### 2.5.4. Determination of Nitrotyrosine

Nitrotyrosine concentration was determined by ELISA using our own rabbit anti-nitrotyrosine polyclonal antibodies (Immunogen: peroxynitrite-modified keyhole limpet hemocyanin), which were coated on an ELISA plate after purification by affinity chromatography within the column with peroxynitrite-treated bovine serum albumin (BSA). After a 2-hour incubation with the specimens, the plate was washed and biotin-labelled murine anti-nitrotyrosine monoclonal antibody (Cayman Chemicals Company, cat. no. 10006966) was added. The dilution of the working solution was 1 : 1000. After another incubation with peroxidase-labelled streptavidin (DakoCytomation, Denmark), the 3,3′,5,5′-Tetramethylbenzidine (TMB) Liquid Substrate System for ELISA (TMB Supersenstive, Sigma-Aldrich, USA) was added. After 20-30 minutes, 0.5 M sulphuric acid solution was added as a stop solution. The absorbance was measured using the Power Wave XS microplate reader (BioTek, USA), and data was processed using the KC Junior software bundle (BioTek, USA). Peroxynitrite-treated rabbit serum albumin (RSA) was used as a standard. The nitrotyrosine content was determined using spectrophotometry based on the molar absorption coefficient of 4300 M^−1^ cm^−1^ [14]. Peroxynitrite was obtained as the product of the H_2_O_2_, NaNO_2_, and HCl reaction with subsequent addition of NaOH solution [15]. The ELISA calibration curve was within the nitrated RSA concentration range of 0-1000 nmol/L, while the within-run coefficient of variation was 4.6%. The serum nitrotyrosine results were expressed in nmol/L; liver nitrotyrosine results were expressed in nmol/g protein.

### 2.6. Statistical Analyses

Statistical analyses were performed using STATISTICA 13 PL (Tibco Inc., Palo Alto, CA, USA), StataSE 12.0 (StataCorp LP, TX, U.S.), and R software (CRAN). The *p* value below 0.05 was considered significant for all comparisons. All tests were two-tailed. Imputations were not done for missing data. Nominal and ordinal data were expressed as percentages, while interval data were expressed as the mean value ± standard deviation if normally distributed, or as median/interquartile range if the distribution was skewed or nonnormal. Distribution of variables was evaluated by the Shapiro-Wilk test, and homogeneity of variances was assessed using Levene's test. One-way parametric ANOVA with the Tukey post hoc test was used for all comparisons.

## 3. Results

For rats exposed to epoxysterols and low-cholesterol feed, there was no effect of dietary intake on serum total cholesterol and LDL- and HDL-cholesterol levels. There was no significant between-group difference in the levels of the above markers. However, triglyceride concentrations were significantly higher in the ECh group than in controls. Serum total phospholipid levels were significantly lower in the ECh group than in controls. Furthermore, there was a significant difference between ECh and ES groups in total phospholipid levels (Table 1).

Liver homogenate analysis demonstrated significant differences in total cholesterol levels between the ES and C groups (*p* < 0.05). There were no significant between-group differences in triglyceride levels in liver homogenates (Table 2).

The analysis of serum inflammatory cytokine (IL-1*β*, IL-6, and TNF-*α*) levels demonstrated that exposure to epoxysterols induces their biosynthesis. A significant increase from baseline in serum IL-1*β* and TNF-*α* levels was shown in both ECh and ES groups. The IL-6 levels were significantly higher in the ECh group as compared to the ES group and controls. Furthermore, the evidence of increased liver production of TNF-*α* was demonstrated in both groups exposed to oxysterols (Table 3).

Serum nitrotyrosine levels were significantly higher in both oxysterol-exposed groups (ECh and ES) than in controls (C). However, only the ECh group had significantly higher nitrotyrosine levels in liver homogenate (Table 4).

## 4. Discussion

There is ample evidence to support extensive and multidirectional effect of oxycholesterol biological activity, whereas their mechanism of action has not been fully understood to date. However, experimental studies in animals exposed to oxycholesterol-enriched feed provide contradictory data on the potential effect of oxycholesterols on the development of atherosclerosis [16–18], although most studies confirm atherogenic effect of oxidized cholesterol derivatives, in particular cholestantriol.

On the other hand, there are significantly fewer studies on the effect of oxyphytosterols on lipid metabolism and development of atherosclerosis in animals. The effect of oxidized phytosterols on lipid metabolism in experimental animals suggests that it is possible to regulate triglyceride and cholesterol concentrations on multiple levels, e.g., by regulating the endogenous lipid synthesis, mediating gastrointestinal lipid absorption, or altering lipid catabolism. For instance, the ability of 5-campestenone to activate *β*-oxidation pathway enzymes and simultaneously inhibit the enzymatic mediators of fatty acid synthesis, by activating the PPAR-*α* receptor and other mechanisms, has been demonstrated [19, 20]. It was capable of lowering serum triglyceride levels in rodent serum [21] and liver [19], which we did not confirm in our study. Bang et al. compared the effect of oxyphytosterols obtained by thermal oxidation of phytosterols and oxysterols (oxidized cholesterol derivatives) on serum and liver triglyceride levels in mice, finding a similar effect of both compounds on the above parameters. He demonstrated a reduction of the serum triglyceride level with no change of its liver content [21]. Similarly, hypolipidemic effect of 24-ethylcholest-4-en-3-one on experimental animals has also been reported [22]. Furthermore, meta-analyses concerning the effects of nonoxidized plant sterols and stanols on the lipid concentrations in human blood indicate their potential for lowering serum triglyceride levels [23, 24]. Considering the above, it is unclear whether the ability to reduce triglyceride levels is a common characteristic shared by all oxidized and nonoxidized sterols or whether it results from contamination of sterol- and stanol-enriched food products with sterol and stanol oxidation products.

In our experiment, we observed different effects of oxysterols: in the ECh group, the serum triglyceride levels increased alongside decreased serum phospholipid levels, with no difference in triglyceride content in the liver. The liver cholesterol content was significantly higher only in the ES group. The observed changes can be partially explained by the dietary induction of acute phase reaction, which may be one of the causes for the increased production of triglyceride-rich lipoproteins in the liver and their elimination from plasma [25]. The concomitant decrease in serum phospholipid levels may additionally promote the development of atherosclerosis. Being relatively phospholipid-rich, lipoproteins such as the HDL are good cholesterol acceptors and the decrease in HDL phospholipid content may inhibit the reverse cholesterol transport from peripheral tissues back to the liver. A decreased phospholipid/cholesterol ratio was found in plasma lipoproteins, erythrocyte membranes, hepatocytes, and endothelial cells as a typical metabolic abnormality in atherosclerosis [26].

The excessive hepatic accumulation of cholesterol may also be explained by the effect of hyperhomocysteinemia on its endogenous biosynthesis, that is, an increased expression of HMG-CoA reductase in hepatocytes, activation of the mevalonate pathway, and resultant excessive cholesterol synthesis [27]. Additional exposure of animals to oxycholesterols or oxyphytosterols may exacerbate endothelial dysfunction, increasing homocysteinemia, which additionally intensifies the cholesterol biosynthesis in the liver.

The analysis of the effect of oxyphytosterols on the development of atherosclerosis in Apo E knockout mice [28] did not show any differences in serum and aortic tissue cholesterol levels between the group exposed to oxysterols and the group receiving only animal fat-rich feed. Additionally, in the group of mice fed with oxysterols, oxidative stress measured with 8-iso-prostaglandin F2*α* (8-iso-PGF2*α*) concentration was significantly increased.

Chronic inflammation of the arterial wall mediated by cytokines, oxidative-modified lipoproteins, and other endothelium-damaging factors is one of the most important mechanisms accelerating the development of atherosclerosis. The majority of proinflammatory cytokines show pleiotropic effects, affecting various cells, including those involved in the development of atherosclerosis and chronic systemic inflammatory response seen in atherosclerosis [29, 30]. Local cytokine production is responsible for extracellular matrix overproduction, growth and proliferation of vascular wall cells, recruitment of vascular luminal cells, endothelial dysfunction, and other processes, which ultimately determine the development of atherosclerosis and its progression rate [30].

The studies of animal models of atherosclerosis demonstrated a marked increase of IL-6 synthesis in response to dietary cholesterol intake [31–33]. Although the data on the effect of oxysterols on cytokine biosynthesis is quite fragmented, it seems to support the oxysterol capability to induce inflammation by causing imbalance between anti-inflammatory and proinflammatory cytokines [34, 35].

The analysis of IL-6 concentrations determined in our experiment indicates that its biosynthesis is mediated by exogenous oxycholesterols, whereas the effect was only significant in the ECh group. However, the elevated IL-6 levels in the ECh group did not affect the C-reactive protein (CRP) levels (unpublished data).

The *tumor necrosis factor α* (*TNF-α*) is a pleiotropic proinflammatory cytokine produced by various cell types, e.g., macrophages, leukocytes, vascular endothelial cells, adipocytes, smooth muscle cells, and cardiomyocytes [25, 36]. It affects the local immune activation by increasing the production of IL-1 and IL-6, thus stimulating endothelial cells to synthesize and release various adhesion molecules (ICAM-1, VCAM-1, and P- and E-selectins). Furthermore, it shows a procoagulant effect and, alongside IL-1 and IL-6, an antifibrinolytic effect [36]. Furthermore, TNF-*α* stimulates macrophages to release metalloproteinases, which leads to accelerated degradation and destabilization of the fibrous cover of atherosclerotic plaques, triggering dyslipidemia, insulin resistance, and endothelial dysfunction.

The analysis of changes in serum TNF-*α* levels in rats during exposure to epoxycholesterols and epoxyphytosterols indicates that it is one of the key variables to differentiate between the groups, as it increased significantly in both ECh and ES groups unlike in controls. Similarly, we observed a significant increase in TNF-*α* production in the liver in animals exposed to oxidized sterols (ECh and ES groups). In the light of the published data, the observed changes in TNF-*α* expression confirm the development of systemic and local inflammation (liver). It has been demonstrated that the diet activates a number of signalling pathways involved in lipid metabolism (SREBP-1) and inflammatory response (PPAR*γ*), as well as TNF-*α*, INF*γ*, TGF*β*1, and PDGF gene expression in Apo E/Leiden 3 transgenic mice fed with cholesterol-rich feed [37]. In line with the above, Ferre et al. demonstrated an increased expression of the IL-1*α* gene in the liver and decreased expression of TNF-*α* receptors (TNF-*α* receptor subfamily member 6) in Apo E-/- mice [38]. It was also shown that by activating liver X-receptor (LXR), 22(R)-hydroxycholesterol significantly increases TNF-*α* mRNA expression and TNF-*α* protein production in cytosol of human hepatocytes [39].

The increase in TNF-*α* production observed in animals exposed to oxysterols may be one of the factors, which exacerbate dyslipidemia, i.e., the increase in serum triglyceride levels in the ECh group and increase in liver cholesterol content. Its mechanism involves increased hepatic fatty acid biosynthesis by TNF-*α* and their release from the adipose tissue as well as impaired clearance of triglyceride-rich lipoproteins (mainly VLDL) from plasma [25]. The TNF-*α* stimulates lipolysis in adipose tissue by activating JNK and p44/42 kinases via increased expression of hormone-sensitive lipase (HSL) and adipose triglyceride lipase (ATGL), without altering their core activity or perlipin expression [25, 40, 41]. Another mechanism of TNF-*α* action seen in rodents involves inhibiting the expression of G protein-coupled receptors (GPCRs) present on the surface of adipocytes, which in turn inhibits the antilipolytic effect of adenosine [42]. The associated increase in plasma-free fatty acid concentrations is considered one of the major contributors of insulin resistance and disorders of carbohydrate metabolism as well as hypertriglyceridemia and metabolic syndrome.

Increased liver biosynthesis of fatty acids mediated by TNF-*α* and other proinflammatory cytokines (e.g., IL-1 and IL-6) manifests as increased endogenous triglyceride-rich lipoprotein levels (mainly VLDL). Its mechanism involves upregulated hepatic synthesis of citrate, an allosteric carboxylase activator of Acetyl-CoA (acetyl-coenzyme A), which is the key enzyme in the fatty acid synthesis pathway [43].

The effect of TNF-*α* on cholesterol metabolism, on the other hand, differs significantly between primates and rodents. In rodents, a slow increase in serum cholesterol levels and its liver biosynthesis is observed (as shown in our experiment), whereas in humans and other primates, either no effect of TNF-*α* on plasma levels of total cholesterol and LDL-cholesterol or their reduced plasma levels have been seen. On the other hand, the mechanisms leading to increased TNF-*α*-mediated cholesterol levels in rodents include increased activity of HMG-CoA reductase and decreased activity of squalene monooxygenase in the liver [44, 45].

The proatherogenic effect of IL-1 includes T- and B-cell stimulation, increased proliferation and differentiation of neutrophils and monocytes, stimulating the expression of adhesive molecules (ICAM-1, VCAM-1), increased vascular endothelial permeability, stimulation of smooth muscle proliferation, and extracellular matrix synthesis [46]. The studies in animal models of atherosclerosis demonstrated an increased expression of IL-1*α* and IL-1*β* mRNAs in the aortas of animals fed with cholesterol. Similarly, their expression in the arterial wall was detectable in immunohistochemical studies [47]. The in vitro exposure of human macrophages or HUVEC cells to oxycholesterols led to an increased IL-1*β* production [48]. It is likely that a similar effect may also occur during the in vivo exposure to oxysterols, as seen in our experimental studies, where exposure to both epoxycholesterols and epoxyphytosterols resulted in a marked increase in serum IL-1*β* concentration, which was more pronounced in the ECh than ES group. The 3-nitrotyrosine, a product of interaction of tyrosine residues with peroxynitrite, plays a special role in oxidative stress-mediated protein damage. The formation of peroxynitrite, a highly reactive molecule, has been demonstrated in a number of chronic inflammatory conditions and cases of perfusion-reperfusion injury. The cells capable of releasing peroxynitrite include vascular endothelial cells, monocytes/macrophages, and neutrophils [49]. Peroxynitrite modifies both extracellular (e.g., plasma) and intracellular (e.g., antioxidant enzymes) proteins, especially the superoxide dismutase (SOD) [50]. Increased protein nitration, e.g., plasminogen or tPA, correlates with impaired coagulation and fibrinolysis, e.g., in diabetic patients [51], whereas an increased nitrotyrosine level may be an independent cardiovascular risk factor [52]. Immunohistochemical studies confirmed the presence of 3-nitrotyrosine residues in the vascular wall in atherosclerosis [53]. Other proteins particularly susceptible to in vivo nitration and chlorination are apolipoproteins, especially Apo A1, present in HDL particles [54]. It has been demonstrated that their exposure to peroxynitrite (also referred to as nitrosative stress) changes the properties of HDL leading to the loss of its antioxidant effect and capability of reverse cholesterol transport. An increased myeloperoxidase activity is an additional factor, which intensifies HDL nitration [54, 55].

The studies assessing changes in nitrated protein levels in animal models of atherosclerosis or hypercholesterolemia are very scarce and include mainly the results of nitrotyrosine determination in plasma and organ proteins of transgenic mice (Apo E-/-; iNOS-/-, or Apo E-/-). The iNOS gene deficiency very significantly inhibited the synthesis of nitrated proteins and slowed the progression of atherosclerosis [56]. Rat exposure to feed containing 2% of cholesterol led to an increase in serum 3-nitrotyrosine levels by over 100%, compared to animals receiving standard feed [57]. Similarly, experimentally induced hypercholesterolemia resulted in increased 3-nitrotyrosine synthesis in brains of rabbits [58]. Our experiment demonstrated that animal exposure to 5*α*,6*α*-epoxycholesterol or epoxyphytosterols in low-cholesterol feed leads to an increased 3-nitrotyrosine synthesis (increased serum nitrated proteins). Nevertheless, the 3-nitrotyrosine content in the liver tissue was significantly elevated only in the ECh group.

Comparing the results of most experimental animal studies with the oxysterol-associated risk in humans, it should be noted that the daily dietary intake of oxidized cholesterol derivatives by experimental animals in our experiment and in other studies reached 10 mg/kg body weight [59], which would translate into a daily intake of about 175-700 mg of oxysterols and represents at least 100% of the average daily cholesterol intake in an adult. Therefore, it is highly unlikely that a diet of a modern individual can comply with these assumptions. Another issue is interspecies differences between experimental animals (usually rabbits, rodents, or birds) and humans.

The limitations of the current study include a small sample size and the inability to monitor the dynamics of changes in the studied parameters, as the inflammatory response changes rapidly throughout the exposure to the studied compounds. Similarly, our results warrant further studies on the effect of other phytosterols and cholesterol derivatives in animal models.

## 5. Conclusion

Rat exposure to 5*α*,6*α*-epoxycholesterols or epoxyphytosterols increases nitrosative stress (increased production of 3-nitrotyrosine) and proinflammatory cytokine synthesis (TNF-*α*, IL-1*β*, and IL-6). This leads to secondary disorder of lipid metabolism in animals.

## Figures and Tables

**Table 1:** Serum lipid levels (mean value ± standard deviation (SD)) in rats exposed to 5*α*,6*α*-epoxycholesterol (ECh group) and 5*α*,6*α*-epoxyphytosterols (ES group) vs. controls (C group).

	ECh group	ES group	C group	*p*
Total cholesterol (mg/dL)	51.6 ± 7.6	52.8 ± 7.5	47.1 ± 6.3	NS
Triglycerides (mg/dL)	58.3 ± 12.4	52.7 ± 10.0	44.9 ± 10.7	<0.01
Phospholipids (mg/dL)	78.6 ± 9.3^∗^	88.5 ± 10.1	89.3 ± 9.2	<0.01
HDL-cholesterol (mg/dL)	22.2 ± 3.6	25.1 ± 4.3	25.0 ± 2.8	NS
HDL-cholesterol (%TCh)	44.0 ± 9.9	48.6 ± 12	53.5 ± 6.8	NS
LDL-cholesterol (mg/dL)	17.7 ± 7.9	17.2 ± 9.6	13.2 ± 5.3	NS

%TCh: total cholesterol; ^∗^*p* < 0.05 vs. ES group; NS: nonsignificant.

**Table 2:** Total cholesterol and tr**i**gl**y**ceride levels (mean value ± standard deviation (SD)) in liver homogenates of rats exposed to 5*α*,6*α*-epoxycholesterol (ECh group) and 5*α*,6*α*-epoxyphytosterols (ES group) vs. controls (C group).

	ECh group	ES group	C group	*p*
Total cholesterol (mg/g tissue)	2.46 ± 0.21	2.58 ± 0.33^∗^	2.32 ± 0.17	<0.05
Triglycerides (mg/g tissue)	22.7 ± 3.0	20.6 ± 3.7	21.2 ± 3.2	NS

^∗^ *p* < 0.05 vs. controls (C); NS: nonsignificant.

**Table 3:** Inflammatory cytokine (TNF-*α*, IL-1*β*, and IL-6) levels (mean value ± standard deviation (SD)) in serum and liver homogenates of rats exposed to 5*α*,6*α*-epoxycholesterol (ECh group) and 5*α*,6*α*-epoxyphytosterols (ES group) vs. controls (C group).

	ECh group	ES group	C group	*p*
Serum TNF-*α* (pg/mL)	43.3 ± 10.6	36.4 ± 9.5	22.8 ± 5	<0.001
Serum IL-1*β* (pg/mL)	4.9 ± 2.7	4.4 ± 2	1.1 ± 1.4	<0.001
Serum IL-6 (pg/mL)	40.4 ± 9.5	26.9 ± 9.1	20.5 ± 6.1	<0.001
Liver TNF-*α* (pg/mg protein)	284.1 ± 45.2	288.2 ± 33.1	208.1 ± 23.3	<0.001

**Table 4:** Nitrotyrosine levels in serum and liver homogenates of rats exposed to 5*α*,6*α*-epoxycholesterol (ECh group) and 5*α*,6*α*-epoxyphytosterols (ES group) vs. controls (C group). The values were expressed as the mean ± standard deviation (SD).

	ECh group	ES group	C group	*p*
Serum nitrotyrosine (nmol/L)	60.5 ± 13.4	47.7 ± 11.5	34.3 ± 8.4	<0.001
Liver nitrotyrosine (nmol/g protein)	119.3 ± 24.1	94.9 ± 21.0	98.4 ± 8.4	<0.01

## Data Availability

All data is included in the tables within the article.
